# Automation bias in AI-assisted detection of cerebral aneurysms on time-of-flight MR angiography

**DOI:** 10.1007/s11547-025-01964-6

**Published:** 2025-02-12

**Authors:** Su Hwan Kim, Severin Schramm, Evamaria Olga Riedel, Lena Schmitzer, Enrike Rosenkranz, Olivia Kertels, Jannis Bodden, Karolin Paprottka, Dominik Sepp, Martin Renz, Jan Kirschke, Thomas Baum, Christian Maegerlein, Tobias Boeckh-Behrens, Claus Zimmer, Benedikt Wiestler, Dennis M. Hedderich

**Affiliations:** Institute of Diagnostic and Interventional Neuroradiology, Klinikum rechts der Isar, School of Medicine and Health, Technical University of Munich, Munich, Germany

**Keywords:** Artificial intelligence, Automation bias, Cerebral aneurysm, MR angiography

## Abstract

**Purpose:**

To determine how automation bias (inclination of humans to overly trust-automated decision-making systems) can affect radiologists when interpreting AI-detected cerebral aneurysm findings in time-of-flight magnetic resonance angiography (TOF-MRA) studies.

**Material and Methods:**

Nine radiologists with varying levels of experience evaluated twenty TOF-MRA examinations for the presence of cerebral aneurysms. Every case was evaluated with and without assistance by the AI software © mdbrain, with a washout period of at least four weeks in-between. Half of the cases included at least one false-positive AI finding. Aneurysm ratings, follow-up recommendations, and reading times were assessed using the Wilcoxon signed-rank test.

**Results:**

False-positive AI results led to significantly higher suspicion of aneurysm findings (*p* = 0.01). Inexperienced readers further recommended significantly more intense follow-up examinations when presented with false-positive AI findings (*p* = 0.005). Reading times were significantly shorter with AI assistance in inexperienced (164.1 vs 228.2 s; *p* < 0.001), moderately experienced (126.2 vs 156.5 s; *p* < 0.009), and very experienced (117.9 vs 153.5 s; *p* < 0.001) readers alike.

**Conclusion:**

Our results demonstrate the susceptibility of radiology readers to automation bias in detecting cerebral aneurysms in TOF-MRA studies when encountering false-positive AI findings. While AI systems for cerebral aneurysm detection can provide benefits, challenges in human–AI interaction need to be mitigated to ensure safe and effective adoption.

**Supplementary Information:**

The online version contains supplementary material available at 10.1007/s11547-025-01964-6.

## Introduction

Cerebral aneurysms have an estimated prevalence of 2% and account for up to 85% of non-traumatic subarachnoid hemorrhages (SAH), which are associated with a considerable risk of severe disability and mortality [1]. Early detection of aneurysms allows for timely rupture risk assessment and optimal management, potentially enhancing patient outcomes [2]. While digital subtraction angiography (DSA) remains the gold standard imaging modality, computed tomography angiography (CTA) and time-of-flight magnetic resonance angiography (TOF-MRA) have proven to be reliable noninvasive methods for detecting cerebral aneurysms [3, 4]. Improvements of these diagnostic imaging techniques have further led to an increased detection of unruptured cerebral aneurysms over time [5, 6].

In recent years, numerous studies have explored the use of artificial intelligence (AI) computer-assisted diagnosis (CAD) systems for the detection of cerebral aneurysms in CTA, MRA, or DSA datasets [7]. These included both studies performing a standalone evaluation of an AI CAD system [8–13] and ones applying AI CAD systems as a reader aid [14–18].

Yet, how cognitive biases affect the diagnostic performance of radiologists when interacting with AI CAD systems for cerebral aneurysm detection remains unknown. One such phenomenon that has been described in the context of AI-assisted diagnosis is automation bias, which is the tendency of humans to overly rely on automated decision-making systems [19–23]. For instance, one recent study demonstrated that radiologists are prone to favor even incorrect suggestions from an AI-based mammogram classification system [22]. Notably, automation bias is a major concern in low-prevalence contexts, such as AI-based cerebral aneurysm detection. In such settings, even highly accurate diagnostic tests yield more false-positive than true-positive cases due to the overwhelming number of normal cases being analyzed (also known as false-positive paradox) [7, 24].

Hence, the aim of this study was to determine how automation bias can affect radiologists with varying experience levels when interpreting AI-detected cerebral aneurysm findings in TOF-MRA studies.

## Methods

Ethical approval was obtained, and the need for informed consent was waived by the Institutional Review Board of the Technical University of Munich.

### Dataset and AI CAD system

The dataset consisted of a total of twenty 3D TOF-MRA studies acquired between 06/2021 and 12/2023 at a collaborating outpatient radiology practice (“Die Radiologie”, Munich, Germany) at which a CAD AI system (© mdbrain, version 4; Mediaire GmbH) for cerebral aneurysm detection is routinely used. TOF-MRA images with segmentations of AI-detected findings were automatically generated by the AI system. Details regarding the training of the AI model have been reported previously [25]. Image studies had been acquired using two clinical 3T scanners (Skyra, Siemens Healthineers, Erlangen, Germany; Ingenia Elition X, Philips Healthcare, Best, The Netherlands) and two 1.5T scanners (Magnetom Aera, Siemens Healthineers, Erlangen, Germany; Ingenia, Philips, Best, Netherlands). Local routine protocols were applied, with the slice thickness ranging between 0.6 and 1.4 mm.

To identify both cases with true-positive and false-positive AI findings, a retrospective full-text search was performed within the radiology information system (RIS). The final dataset included ten cases with at least one false-positive AI finding and ten cases with at least one true-positive AI finding but no incorrect AI findings. In total, the dataset included twelve aneurysms (eleven saccular and one fusiform aneurysm). Only findings of the anterior circulation were considered for the purpose of this study. Cases were selected and verified by a board-certified interventional neuroradiologist.

TOF-MRA studies were independently reviewed by two senior neuroradiologists (16 years and 9 years of neuroradiology experience each) for the presence of cerebral aneurysms. Full consensus was reached between the two neuroradiologists in all twenty cases. Digital subtraction angiography (DSA) was only available in a single case. False-positive AI findings were classified as vascular loop (5/10), infundibulum (3/10), or perforator (2/10) (Supplement 1).

### Reader study

Anonymized image datasets with and without AI annotations were imported to our local Picture Archiving and Communicating System (PACS) system (IDS7, Sectra Medical Systems AB, USA). Cases were randomized into two sets (A and B), each containing four and six false-positive cases.

**Fig. 1:**
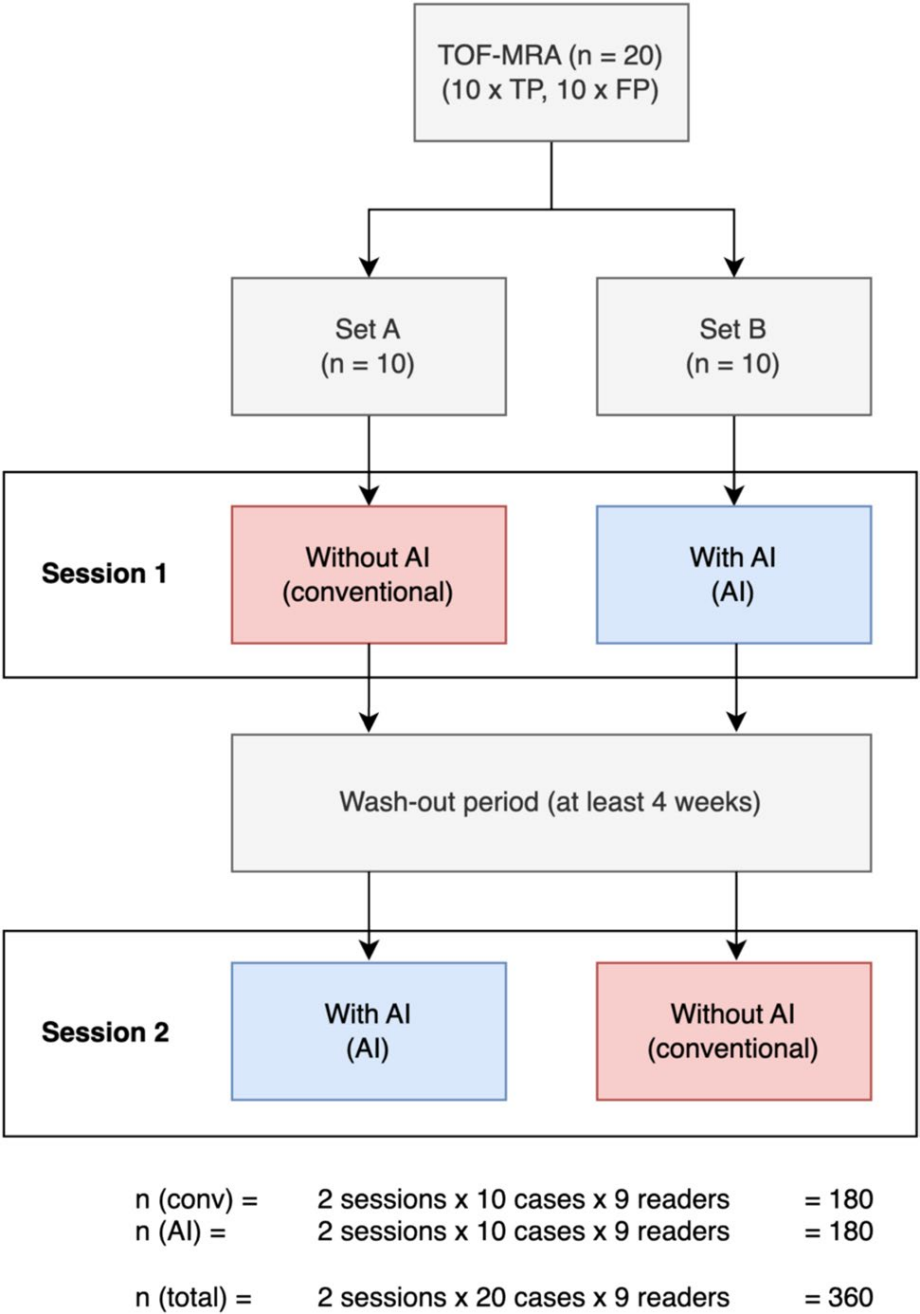
Study Design. A total of nine readers evaluated the dataset twice, with a washout period of at least four weeks between the two sessions. In each session, one set of cases was evaluated with AI assistance and the other without

A total of nine readers evaluated the dataset twice, with a washout period of at least four weeks between the two sessions (average: 45 days) (Fig. 1). Participants included three inexperienced (radiology residents with 6–12 months of neuroradiology experience), three moderately experienced (board-certified radiologists), and three very experienced readers (board-certified neuroradiologists) (Table 1). Crucially, readers were blinded to the study design and the composition of the study cohort. In each session, one set of cases was evaluated with AI assistance and the other without. Before the first session, five sample cases with verified true-positive AI findings were showcased to familiarize the readers with the AI annotations and cultivate trust in the tool’s accuracy. To define a clear interaction protocol, readers were instructed to examine AI annotations before reviewing the original TOF-MRA image series (AI as “first reader”). By default, only the axial source TOF-MRA series (with and without AI annotations) were included in the hanging protocol, but readers were allowed to perform multiplanar and 3D reconstructions as well as maximum intensity projections (MIP) at will.

**Table 1:** Overview of readers

reader_id	Experience level	Age range	Sex	Radiology experience (in years)	Neuroradiology experience (in years)	Experience in neuroradiology interventions (in years)
E1R1	Inexperienced	26–30	Female	1	1	0
E1R2	Inexperienced	26–30	Female	0.5	0.5	0
E1R3	Inexperienced	26–30	Female	0.5	0.5	0
E2R1	Moderately experienced	31–35	Female	6.5	2.5	2
E2R2	Moderately experienced	31–35	Male	6	2	1
E2R3	Moderately experienced	36–40	Female	5	3	1
E3R1	Very experienced	41–45	Male	8	6	4
E3R2	Very experienced	36–40	Male	9	4	3
E3R3	Very experienced	46–50	Male	18	14	10

For each arterial segment of the anterior circulation, readers provided a 4-point Likert-scale rating on the presence of a cerebral aneurysm integrating diagnostic evaluation and certainty into a single variable (1: certainly not present, 2: likely not present, 3: likely present, 4: certainly present). In addition, for each aneurysm (not each patient), readers indicated a follow-up recommendation (no follow-up examination, MRI, or DSA). The patient age was provided only upon request. Aneurysm ratings and recommendations were documented using an online form tool (© Google Forms, Google Inc., Mountain View, USA), and reading times were recorded using a time tracking software (Toggl Track, © Toggl OÜ, Tallinn, Estonia).

### Sample size calculation

Given that this study compared the diagnostic evaluation of radiologists with and without AI assistance, the sample size was defined as the number of readings (number of readers x number of cases x number of readings per reader and case). Based on the previous research on the impact of CAD tools for cerebral aneurysm detection [7], a small effect size of 0.2 was assumed. Using a statistical power of 80%, an α error probability of 0.05, and a two-tailed matched-pair Wilcoxon signed-rank test, a minimum sample size of 208 was determined (G*Power, v3.1).

### Analysis

Data manipulation, data visualization, and statistical analyses were performed using Python (version 3.9.7).

To prevent the misclassification of findings due to inaccurate locations, findings with a Likert-scale rating of 2 or more were grouped as ‘Acom (anterior communicating artery)’ if the location was described as ‘A1 segment (anterior cerebral artery)’, ‘Acom’, or ‘A2 segment (anterior cerebral artery)’, and as ‘ICA (internal carotid artery)’ if the location was described as ‘ICA’ or ‘terminal T’. Follow-up recommendations were modeled as an ordinal scale from 0 to 2 (0: no follow-up, 1: MRI, 2: DSA), reflecting the level of diagnostic intensity. The degree of bias toward false-positive AI findings was quantified by comparing aneurysm ratings and follow-up recommendations for false-positive AI findings between the two reading workflows (conventional vs with AI).

Normality of data distribution was evaluated using the Shapiro–Wilk test. The level of statistical significance was set at *p* = 0.05. Accounting for the paired nature of the data (rating of the same findings with or without AI), a Wilcoxon signed-rank test was used to evaluate statistical significance for aneurysm ratings, follow-up recommendations, and reading times.

Sensitivity (per lesion), sensitivity (per patient), and specificity (per patient) are reported, grouped by reading workflows and experience levels. Binary classifications for the presence of aneurysms were inferred from the 4-point Likert-scale ratings (1–2: absent, 3–4: present). The diagnostic classification per patient was performed as follows [18]: A case was classified as true-positive only when the reader identified all aneurysms without reporting any false positives. True negatives were defined as cases where both the reader and the reference standard agreed on the absence of any aneurysms. Cases with at least one false-positive finding were labeled as false positive. Cases where the reader failed to detect a true aneurysm were considered false negatives. Diagnostic accuracy measures with and without AI assistance were compared statistically using the McNemar test.

To illustrate the impact of automation bias, rating pairs for false-positive AI findings (conventional vs with AI) were visualized using Sankey diagrams, grouped by experience level. For individual readers, associations between diagnostic performance metrics, mean reading times, and frequencies of unconfident Likert-scale ratings (2 or 3) were determined using the Spearman’s correlation coefficient. Results were displayed in a color-coded correlation matrix.

A thematic analysis was performed to summarize reader feedback and observations.

## Results

**Fig. 2:**
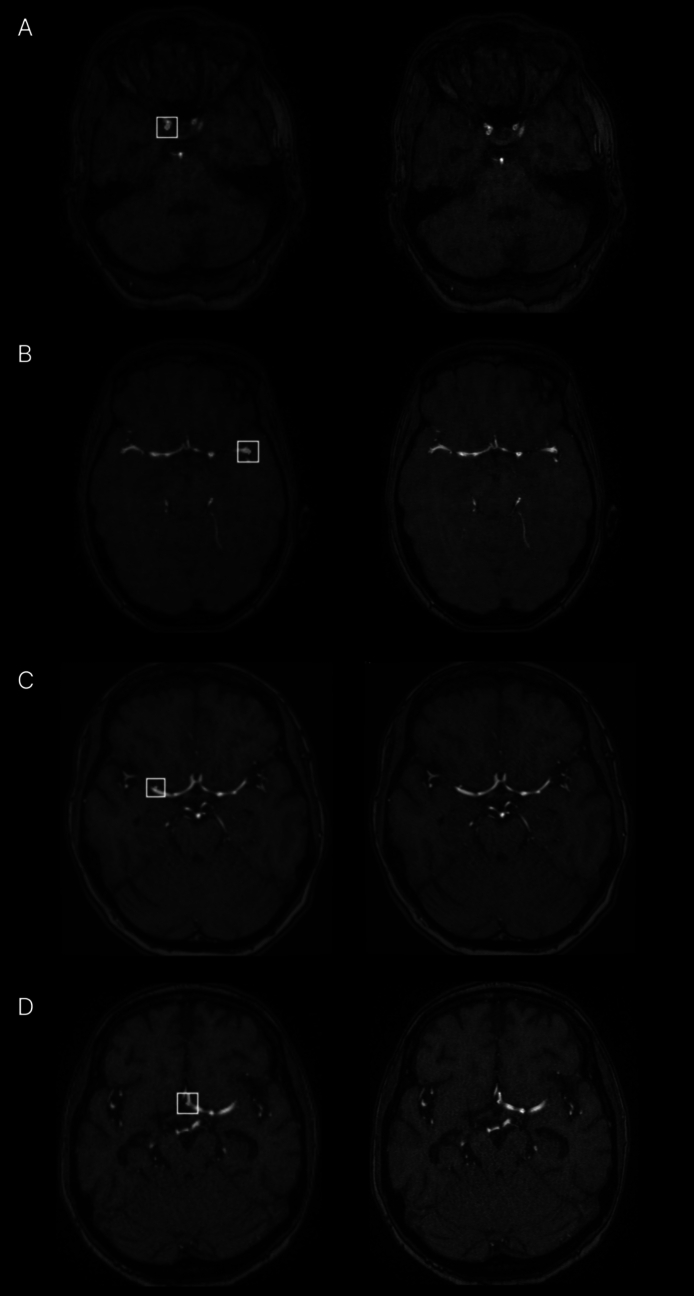
Sample cases. Representative slices from each TOF-MRA scan with (left) and without (right) AI annotations are shown. **A** Aneurysm of the right internal carotid artery (ICA) (true-positive AI finding). **B** Aneurysm of the left middle cerebral artery (MCA) bifurcation (true-positive AI finding). **C** Infundibulum arising from the right M1 segment (false-positive AI finding). **D** Fenestrated anterior communicating artery (Acom) (false-positive AI finding)

The study involved a sample size of 360 readings (2 readings × 9 readers × 20 cases), which exceeded the calculated minimum sample size required for adequate power, as determined by our power analysis. Exemplary TOF-MRA images of true-positive and false-positive cases are shown in Fig. 2.

### Automation bias

**Fig. 3:**
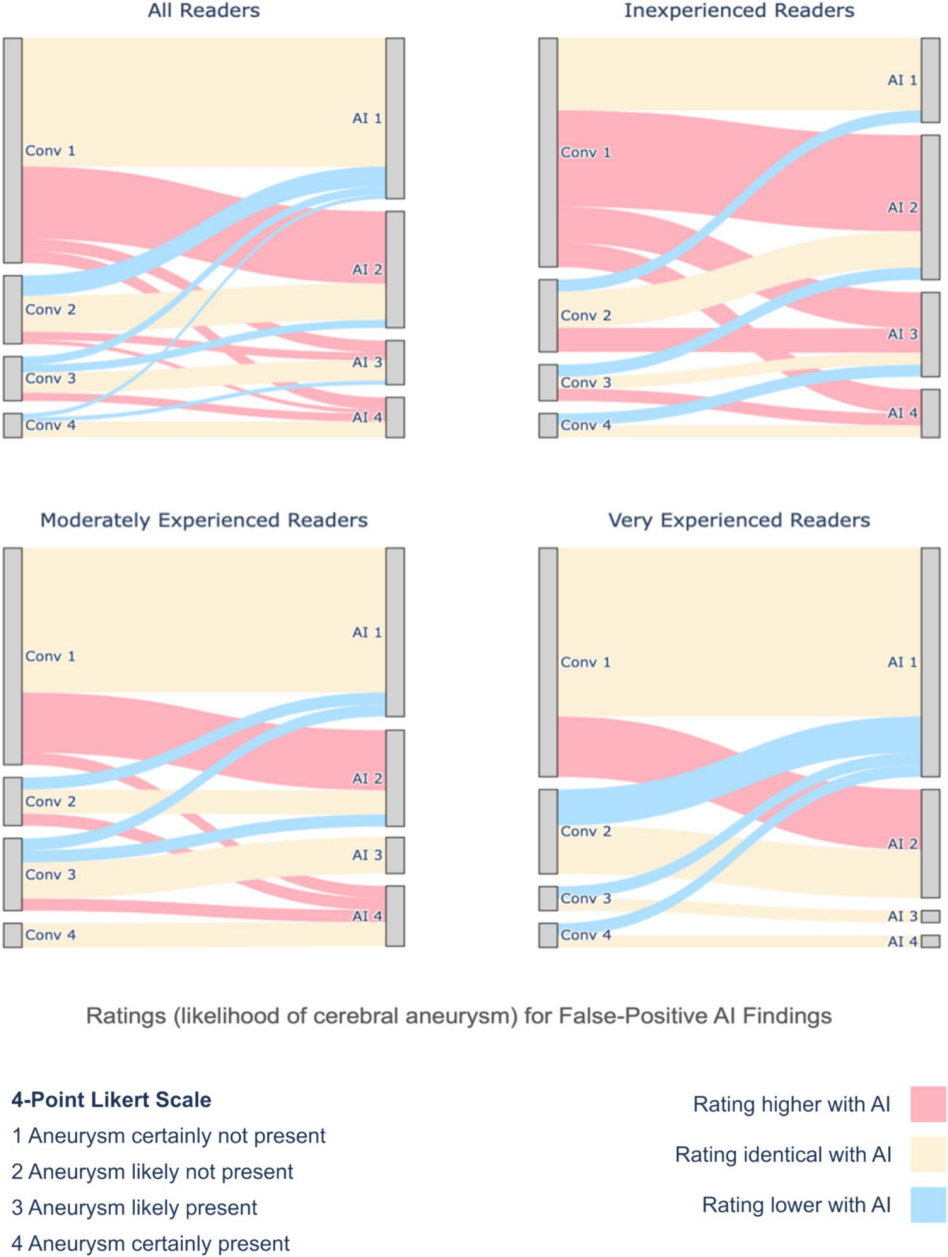
Likert-scale ratings (by workflow and experience level) for false-positive AI findings. The Sankey diagrams illustrate how readers rated identical vascular segments for the presence of aneurysms in the presence (‘AI’) and absence (‘Conv’) of false-positive AI results on a Likert scale from 1–4 (1: certainly absent, 2: likely absent, 3: likely present, 4: certainly present). For instance, ‘Conv 3’ indicates that a vascular segment was rated as ‘aneurysm likely present’ without AI assistance. Connections from left to right nodes are marked red, yellow, or blue depending on whether ratings were higher, identical, or lower with AI assistance than without (conventional). Inexperienced readers were early residents with less than 1 year of neuroradiology experience, moderately experienced readers board-certified radiologists, and very experienced readers certified neuroradiologists

Exposure of readers with false-positive AI recommendations led to significantly higher Likert-scale ratings of suspected aneurysms (*p* = 0.01). Ratings of inexperienced readers were particularly influenced by false-positive AI findings, with case rates as unremarkable (rating of 1) dropping from 63.3% (19/30) to 23.3% (7/30). This shift was accompanied by a significant change across the full range of Likert ratings (*p* = 0.002). In contrast, moderately experienced readers showed a smaller decline in unremarkable ratings (conventional: 60.0% [18/30], AI: 46.7% [14/30]), and no change was seen in very experienced readers (conventional: 63.3% [19/30], AI: 63.3% [19/30]). For both subgroups, changes in Likert ratings were not statistically significant (moderately experienced: *p* = 0.18, very experienced: *p* = 0.59). Most instances where aneurysm ratings increased with AI involved arterial segments that were confidently rated as unremarkable (rating of 1) without AI support (Fig. 3).

### Follow-up recommendations

**Fig. 4:**
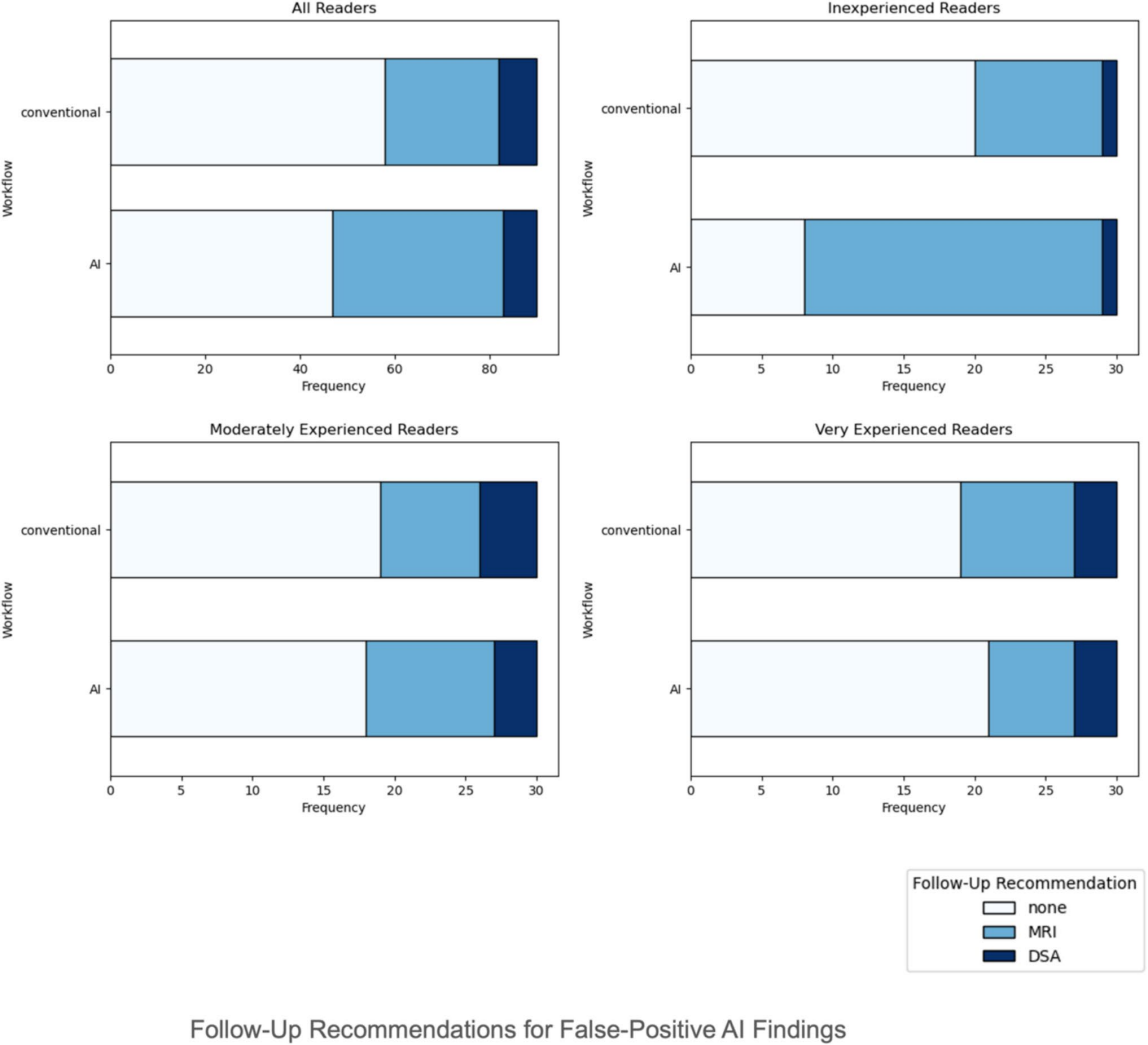
Follow-up recommendations (by workflow and experience level) for false-positive AI findings. Recommendations were modeled as an ordinal scale from 0 to 2 (0: no follow-up, 1: follow-up MRI, 2: digital subtraction angiography; DSA), reflecting the level of diagnostic intensity. ‘Conventional’ indicates the follow-up recommendations provided by readers for arterial segments without knowledge of the false- positive AI result. Significantly more intense follow-up strategies were observed with AI assistance in inexperienced readers (*p* = 0.005), but not in moderately experienced (*p* = 1.0) or very experienced readers (*p* = 0.71)

Follow-up recommendations for false-positive AI findings varied by reader experience (Fig. 4). Overall, readers more frequently recommended MRI and DSA in the AI-assisted workflow (conventional: MRI = 24, DSA = 8; AI: MRI = 36, DSA = 7), although the difference was not significant (*p* = 0.21). Inexperienced readers recommended a significantly more intense follow-up strategy (*p* = 0.005), with a marked increase in MRI recommendations with AI (conventional: MRI = 9; AI: MRI = 21) but consistent DSA recommendations (1 in both workflows). Moderately experienced readers (conventional: MRI = 7, DSA = 4; AI: MRI = 9, DSA = 3; *p* = 1.0) and very experienced readers (conventional: MRI = 8, DSA = 3; AI: MRI = 6, DSA = 3; *p* = 0.71) alike showed no significant changes in follow-up recommendations.

### Interpretation times

**Fig. 5:**
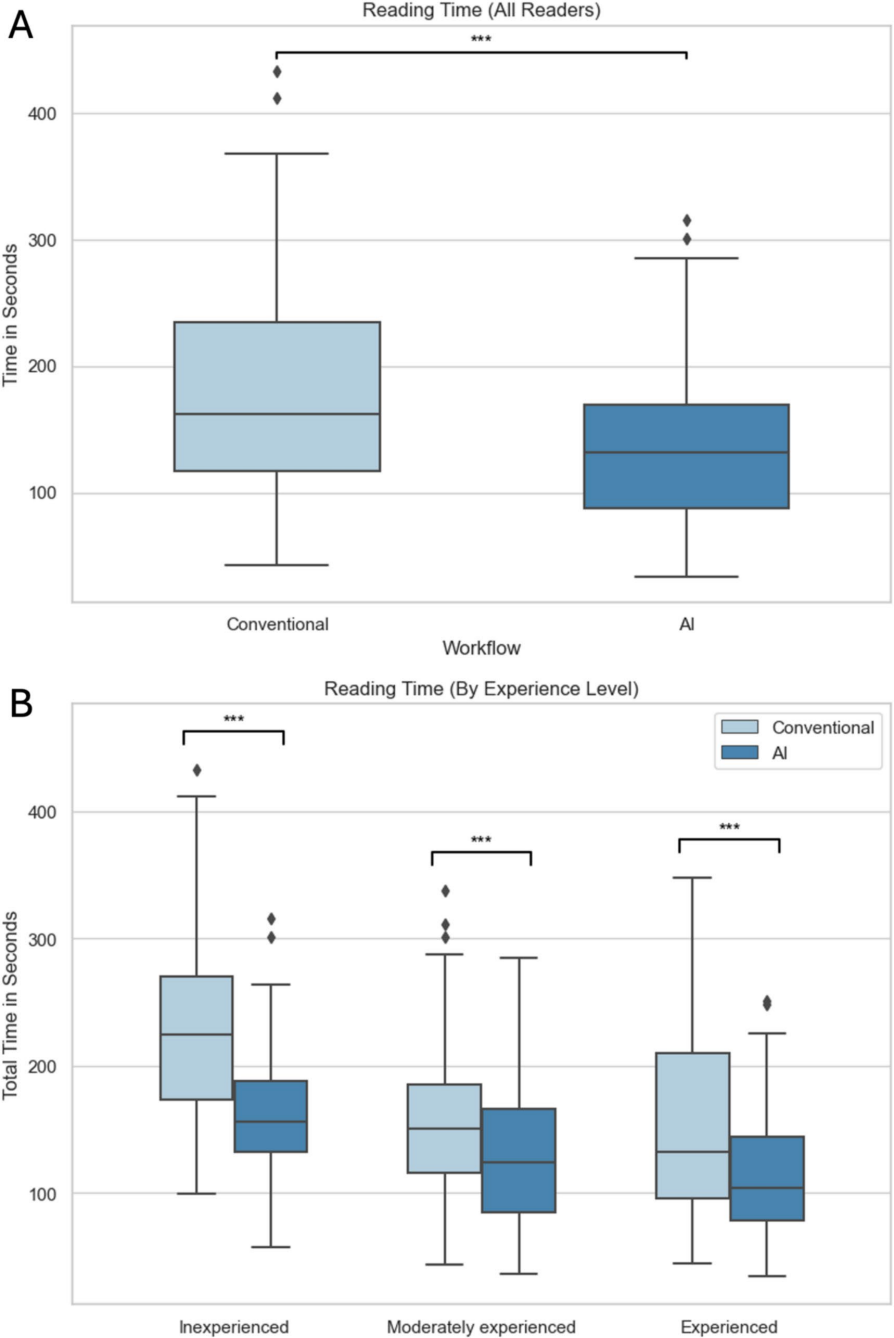
Reading times. **A** Overall. **B** By experience level

Mean reading times were significantly shorter with AI assistance in inexperienced (164.1 vs 228.2 s; *p* < 0.001), moderately experienced (126.2 vs 156.5 s; *p* < 0.009), and very experienced (117.9 vs 153.5 s; *p* < 0.001) readers alike. Overall, mean reading times were reduced significantly from 179.4 to 136.0 s with AI support (*p* <  < 0.001) (Fig. 5).

### Diagnostic performance

Overall, the combined per-lesion and per-patient sensitivity both increased from 0.88 in the conventional workflow to 0.97 with AI assistance (*p* = 0.02 for both). Specificity per patient remained unchanged at 0.79 in both workflows (*p* = 1.00) (Table 2). Inexperienced readers exhibited a significant improvement in per-lesion and per-patient sensitivity with AI (from 0.69 to 1.00 for both, *p* = 0.001), which, however, came at the cost of decreased specificity (conventional: 0.81, with AI: 0.66; *p* = 0.20). Moderately experienced readers showed exceptional per-lesion and per-patient sensitivity in both workflows (consistently at 0.97; *p* = 1.00) and reached comparable specificity with AI assistance (conventional: 0.70, with AI: 0.79; *p* = 0.43). Similarly, very experienced readers displayed very high sensitivity on lesion level (conventional: 0.94, with AI: 0.97; *p* = 1.00) and patient level (conventional: 0.97, with AI: 0.94; *p* = 1.00), while also demonstrating superior specificity over the other reader groups (conventional: 0.88, with AI: 0.94; *p* = 0.70).

**Table 2:** Diagnostic performance by reader group and workflow (all cases). Accuracy metrics were statistically compared using the McNemar’s test

Reader group	Sensitivity (per lesion)			Sensitivity (per patient)			Specificity (per patient)		
	Conventional	With AI	*P*-value	Conventional	With AI	*P*-value	Conventional	With AI	*P*-value
Inexperienced (*n* = 3)	0.69	1.00	0.001	0.69	1.00	0.001	0.81	0.66	0.20
Moderately experienced (n = 3)	0.97	0.97	1.00	0.97	0.97	1.00	0.70	0.79	0.43
Very experienced (n = 3)	0.94	0.97	1.00	0.97	0.94	1.000	0.88	0.94	0.70
Overall (n = 9)	0.88	0.97	0.02	0.88	0.97	0.02	0.79	0.79	1.00

### Feedback and observations

Qualitative findings obtained from reader feedback and observations are summarized in Table 3. There was overwhelming agreement that high sensitivity of the AI tool was far more important than specificity, given that the consequences of missing a true aneurysm are more severe. At the same time, many readers trusted that the commercial AI software must have been optimized for high sensitivity and showed little concern about aneurysms potentially missed by the AI. Most readers appreciated the AI’s utility in filtering out potentially relevant findings that require more careful review by the human reader. The AI assistance was perceived as reassuring, particularly by the inexperienced readers.

**Table 3:** Reader feedback and observations

Theme	Feedback and observations
Diagnostic performance	High sensitivity is more important than specificity, given that consequences of missing an aneurysm could be critical Many readers trusted that AI must have been optimized for high sensitivity
Clinical utility and adoption	Human readers should sort out false-positive findings Particularly useful for distal findings easy to overlook Helps to direct attention to potentially relevant findings False-positive AI findings may artificially create morbidity leading to invasive procedures with potential complications (e.g., DSA)
Psychological aspects	It is reassuring to have an AI double-check findings Due to medicolegal implications, it is difficult to not follow-up on AI-positive findings, even when an aneurysm is considered very unlikely The availability of AI results promotes complacency, radiologists should make a deliberate effort to systematically review all arterial segments even in the presence of an AI assistance Clinical referrers are unsettled when AI-positive findings are reported, radiologists are required to justify contrary opinions In the presence of one aneurysm, very experienced radiologists tend to more carefully look for the presence of further aneurysms (opposite of “satisfaction of search”)

However, readers remarked that the availability of an automated aneurysm detection software promotes complacency and that radiologists should make a deliberate effort to review the images systematically, nonetheless. Interestingly, two radiologists stated that they would feel compelled to follow-up on AI-positive findings due to potential medicolegal repercussions, even when they personally doubted the presence of an aneurysm. One reader noted that referring physicians are equally unsettled when made aware of AI-positive findings, necessitating that radiologists justify any opposing assessments. On a 5-point Likert scale (1: not at all helpful, 5: very helpful), the AI software received a median rating of 4.

### Reader-level correlation

**Fig. 6:**
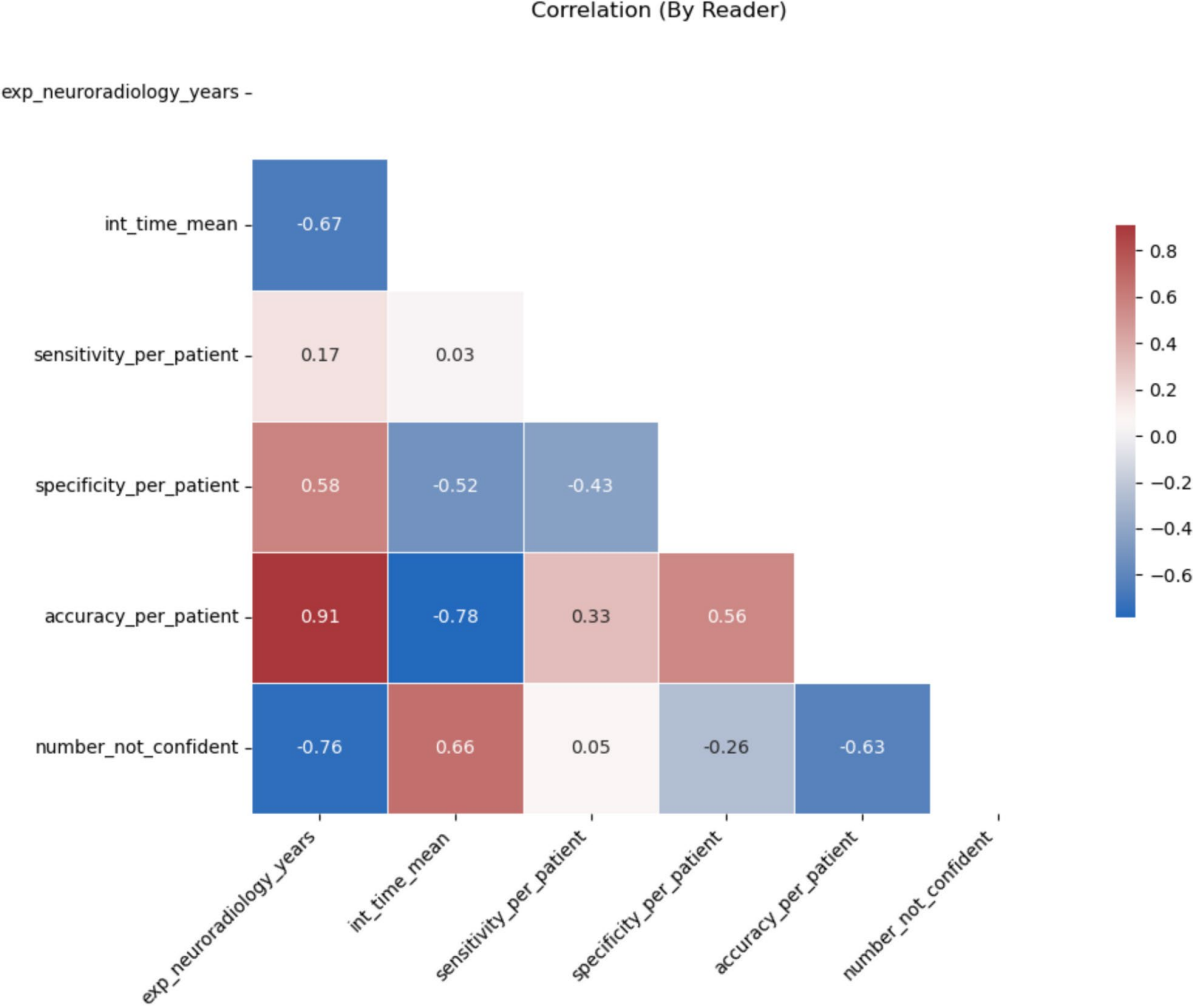
Correlation matrix (by individual reader). The Spearman’s correlation coefficient was calculated to determine the association between diagnostic performance metrics, mean reading times, and frequency of unconfident ratings (Likert-scale rating of 2 or 3). Red and blue colors indicate a positive and negative correlation between variables each. For instance, neuroradiological experience in years shows a strong positive correlation with diagnostic accuracy per patient and a strong negative correlation with number of unconfident ratings

Correlation of reader-specific metrics reveals important associations (Fig. 6). Notably, the level of neuroradiology experience showed a strong positive correlation with per-patient accuracy and a moderate positive correlation with specificity. Experience negatively correlated with mean reading time and number of unconfident ratings (Likert-scale rating of 2 or 3), indicating that experienced readers were faster and had more confidence in their assessments. The number of unconfident ratings positively correlated with mean reading time and negatively correlated with patient-level accuracy, possibly mediated by experience level.

## Discussion

This study explored how automation bias can affect radiologists with varying experience levels when evaluating AI-detected cerebral aneurysm findings in TOF-MRA scans. Radiologists were more likely to believe the presence of an actual aneurysm when presented with false-positive AI findings, demonstrating their susceptibility to automation bias. This observation corroborates previous warnings about the adverse effects of over-reliance on AI tools in medical imaging [26–28]. While inexperienced readers were strongly influenced by the AI findings, very experienced readers demonstrated resilience to this bias. These results are consistent with an earlier study on mammography reading revealing diminishing degrees of automation bias with greater reader experience [22], underlining the critical role of experienced radiologists in validating AI results. Importantly, inexperienced readers were also significantly more likely to recommend follow-up examinations in response to false-positive AI findings. This indicates that incorrect AI suggestions can lead to unnecessary procedures, raising both costs and patient anxiety.

The significance of false-positive findings in the context of AI-based cerebral aneurysm detection can be demonstrated by estimating the frequency distribution of diagnostic outcomes. Assuming a prevalence of 2%, an AI system with a sensitivity of 91.2% and a specificity of 83.5% [7] results in around 16.2% of all AI evaluations being false positive. Moreover, the corresponding positive predictive value of approximately 11.3% implies that only one in nine AI-detected findings represents a true aneurysm (Supplement 2). Consequently, even a small proportion of false-positive AI findings leading to unnecessary follow-up examinations could impose a substantial burden on the healthcare system. Future studies should explore the health economic implications of such redundant medical services caused by erroneous AI findings in depth.

Intriguingly, some of the more experienced readers commented that in actual practice, they might choose to recommend follow-up examinations for AI-positive findings even when an aneurysm appeared improbable, simply to avoid medicolegal risks. Extending earlier concerns about legal liability for errors committed by AI systems [29, 30], this finding suggests that fears of legal repercussions for overriding correct AI results can lead to medically irrational decisions. These concerns are not fully unfounded, considering that certified AI systems in radiology commonly have intended-use statements with disclaimers passing the medicolegal responsibility onto the user [31]. Clear guidelines and legislation are necessary to empower physicians to make decisions based on medical expertise and patient needs, rather than legal concerns.

A recurring concern raised by several readers was the lack of standardized guidelines for the management of cerebral aneurysms. According to the latest 2022 *European Stroke Organisation (ESO)* guidelines, follow-up imaging frequency and duration should be determined "*based on aneurysm- and patient-related risk factors for growth or rupture, and risk of treatment”* [32]. Although this statement reflects the complexity of aneurysm management and the individuality of each patient case, it does not offer practical guidance for radiologists in establishing a consistent follow-up strategy for incidental aneurysms. A 2018 survey study revealed considerable heterogeneity among neuroradiologists in follow-up recommendations for small (< 7 mm) unruptured aneurysms, further underscoring the necessity for standardized protocols [33].

Unlike a previous study that reported mixed effects of AI assistance on TOF-MRA reading times of three radiology readers and three students [18], this study observed decreased reading times across all experience levels. The fact that this efficiency improvement was demonstrated in an artificial cohort with an extraordinarily high overall rate of false-positive AI findings (50%) is noteworthy, since false-positive findings have been suspected to cause increased workload [7].

### Limitations

This study has several limitations.

First, the reference standard was defined by expert consensus based on TOF-MRA images rather than DSA, which is widely regarded as the gold standard modality, but was available in only one case. Nonetheless, the fact that the two experts who defined the reference standard and the most senior reader participating in the study (14 years of neuroradiology experience) had a 100% agreement (without AI), suggests a robust reference standard.

Second, the readings took place in a controlled study setting and readers might behave differently in clinical routine under high workload. It is likely that readers evaluated TOF-MRA scans more thoroughly than usual, as they were explicitly instructed to search for aneurysms.

Third, the case cohort did not have a representative distribution of diagnostic outcomes, limiting the quantification of potential clinical impact. Future studies should evaluate automation bias in a larger, more representative cohort.

Fourth, potential bias toward false-negative AI results was not assessed. With previously reported per-lesion sensitivities of aneurysm detection systems ranging widely from 67 to 100% [7] and given the high reliance of readers on the AI’s sensitivity observed in this study, it is conceivable that false-negative AI results lead to a higher frequency of missed aneurysms.

## Conclusion

Our results demonstrate vulnerability of radiology readers to automation bias in detecting cerebral aneurysms in TOF-MRA examinations when faced with false-positive AI findings. Importantly, this behavior further translated into more intense follow-up recommendations among inexperienced readers. AI assistance resulted in significantly shorter reading times across experience levels, even in our artificial cohort with a very high overall rate of false-positive AI findings. While AI-assisted reading of radiology images can have benefits, challenges in human–AI interaction need to be mitigated to ensure safe and effective adoption.

## Supplementary Information

Below is the link to the electronic supplementary material.

Supplementary file1 (DOCX 155 kb)

## Abbreviations

CAD: Computer-assisted diagnosis
CTA: Computed tomography angiography
TOF-MRA: Time-of-flight magnetic resonance angiography
DSA: Digital subtraction angiography
AI: Artificial intelligence
