# Supplementary material for: Automation bias in AI-assisted detection of cerebral aneurysms on time-of-flight MR angiography
Source: Radiol Med. 2025 Feb 12;130(4):555–66. doi: 10.1007/s11547-025-01964-6 (PMC12008054; doi:10.1007/s11547-025-01964-6)
Supplement: Supplementary file 1 — Supplementary file1 (DOCX 155 kb) [file 11547_2025_1964_MOESM1_ESM.docx]

| case_id | finding_id | Age Range | Evaluation of AI Finding | Diameter of AI Finding (in mm) | Location | Description |
| --- | --- | --- | --- | --- | --- | --- |
| A01 | A01_1 | 41 – 45 | False Positive | 2.6 | Right M1 segment | Infundibulum |
| A02 | A02_1 | 51 – 55 | True Positive | 2.8 | Left MCA bifurcation | Saccular aneurysm |
| A03 | A03_1 | 51 – 55 | True Positive | 3.7 | Right MCA bifurcation | Saccular aneurysm |
| A03 | A03_2 | 51 – 55 | False Positive | 1.7 | Left M1 segment | Infundibulum |
| A04 | A04_2 | 56 – 60 | False Positive | 1.3 | Left M2 segment | Vascular loop |
| A05 | A05_1 | 51 – 55 | True Positive | 4.6 | Right ICA | Saccular aneurysm |
| A06 | A06_1 | 71 – 75 | False Positive | 2.8 | Right ICA | Infundibulum |
| A07 | A07_1 | 66 – 70 | True Positive | 3.9 | Right ICA | Saccular aneurysm |
| A08 | A08_1 | 81 – 85 | True Positive | 2.5 | Acom | Saccular aneurysm |
| A09 | A09_1 | 76 - 80 | True Positive | 4.7 | Acom | Saccular aneurysm |
| A09 | A09_2 | 76 – 80 | True Positive | 3.0 | Right MCA bifurcation | Saccular aneurysm |
| A10 | A10_1 | 71 – 75 | False Positive | 2.9 | Acom | Acom fenestration |
| B01 | B01_1 | 76 – 80 | False Positive | 1.4 | Right ICA | Vascular loop |
| B02 | B02_1 | 16 – 20 | False Positive | 2.2 | Left M2 segment | Vascular loop |
| B03 | B03_1 | 71 – 75 | True Positive | 5.6 | Left MCA bifurcation | Saccular aneurysm |
| B03 | B03_2 | 71 – 75 | True Positive | 3.7 | Left ICA | Saccular aneurysm |
| B04 | B04_1 | 56 – 60 | True Positive | 3.1 | Left MCA bifurcation | Fusiform aneurysm |
| B05 | B05_1 | 31 – 35 | False Positive | 1.5 | Left M1 segment | Perforator |
| B06 | B06_1 | 26 – 30 | False Positive | 2.0 | Left M1 segment | Perforator |
| B07 | B07_1 | 31 – 35 | True Positive | 4.0 | Left ICA | Saccular aneurysm |
| B08 | B08_1 | 26 – 30 | False Positive | 1.2 | Acom | Vascular loop |
| B09 | B09_1 | 56 – 60 | True Positive | 4.0 | Left MCA bifurcation | Saccular aneurysm |
| B10 | B10_1 | 31 – 35 | False Positive | 2.2 | Left MCA bifurcation | Vascular loop |

Supplement 1: Overview of AI-positive findings.

|  | Aneurysm Present | Aneurysm Absent | Predictive Value | Total |
| --- | --- | --- | --- | --- |
| Test Positive | True Positive  1.82% (182/10,000) | False Positive  16.17% (1,617/10,000) | Positive Predictive Value  11.3% (182/1,799) | 1,799 |
| Test Negative | False Negative  0.18% (18/10,000) | True Negative  81.83% (8,183/10,000) | Negative Predictive Value  99.8% (8,183/8,201) | 8,201 |
| Accuracy Metrics | Sensitivity  91.2% (182/200) | Specificity  83.5% (8,183/9,800) |  |  |
| Total | 200 | 9,800 |  | 10,000 |

Supplement 2: Contingency table for AI-based cerebral aneurysm detection. A real-world scenario with 10,000 cases was simulated. A prevalence of 2% was assumed. A sensitivity of 91.2% and specificity of 83.5% were assumed based on a recent meta-analysis (Din et al. 2023). These conditions result in a very low positive predictive value (PPV) of 11.3%, implying that only around one in nine AI-detected findings represents a true aneurysm. Numbers were rounded to the nearest whole number.
